# Metropolitan Hospital Sunday Fund

**Published:** 1895-12-21

**Authors:** 


### METROPOLITAN HOSPITAL SUNDAY FUND.

The annual general meeting of the constituents of the Metropolitan Hospital Sunday Fund was held at the Mansion House on Wednesday, 18th inst., under under the presidency of the Lord Mayor. The attendance was large. The report of the Council for the year 1895 was adopted (a digest of which has already appeared in our columns). The Council for the year 1896 was elected, and the next Hospital Sunday was fixed to be held on June 14th, 1896.

